# oncoNcRNA: A Web Portal for Exploring the Non-Coding RNAs with Oncogenic Potentials in Human Cancers

**DOI:** 10.3390/ncrna3010007

**Published:** 2017-02-07

**Authors:** Ze-Lin Wang, Xiao-Qin Zhang, Hui Zhou, Jian-Hua Yang, Liang-Hu Qu

**Affiliations:** 1 Key Laboratory of Gene Engineering of the Ministry of Education, GuangZhou 510275, China; wangzl6@mail2.sysu.edu.cn; 2 State Key Laboratory for Biocontrol, Sun Yat-sen University, Guangzhou 510275, China; wangzl6@mail2.sysu.edu.cn; 3 School of medicine, South China University of Technology, Guangzhou 510640, China; zhxq@connect.hku.hk

**Keywords:** ncRNAs, cancer, somatic copy number alteration, biomarker

## Abstract

Non-coding RNAs (ncRNAs) have been shown to contribute to tumorigenesis and progression. However, the functions of the majority of ncRNAs remain unclear. Through integrating published large-scale somatic copy number alterations (SCNAs) data from various human cancer types, we have developed oncoNcRNA, a user-friendly web portal to explore ncRNAs with oncogenic potential in human cancers. The portal characterizes the SCNAs of over 58,000 long non-coding RNAs (lncRNAs), 34,000 piwi-interacting RNAs (piRNAs), 2700 microRNAs (miRNAs), 600 transfer RNAs (tRNAs) and 400 small nucleolar RNAs (snoRNAs) in 64 human cancer types. It enables researchers to rapidly and intuitively analyze the oncogenic potential of ncRNAs of interest. Indeed, we have discovered a large number of ncRNAs which are frequently amplified or deleted within and across tumor types. Moreover, we built a web-based tool, Correlations, to explore the relationships between gene expression and copy number from ~10,000 tumor samples in 36 cancer types identified by The Cancer Genome Atlas (TCGA). oncoNcRNA is a valuable tool for investigating the function and clinical relevance of ncRNAs in human cancers. oncoNcRNA is freely available at http://rna.sysu.edu.cn/onconcrna/.

## 1. Introduction

It has been clear that the majority of the human (or mammalian) genome is transcribed as non-coding RNAs (ncRNAs) such as well-studied miRNAs and long non-coding RNAs (lncRNAs) [1,2,3,4,5,6,7,8,9]. The most recent study has identified more than 58,000 lncRNA genes in the human genome [10]. However, only a small portion of lncRNAs have been characterized. Similarly, for other ncRNA classes like small nucleolar RNA (snoRNA), piwi-interacting RNA (piRNA) and transfer RNA (tRNA), whether they can contribute to carcinogenesis also remains unclear.

Somatic copy number alterations (SCNAs) are extremely common in cancer [11,12], which can promote tumorigenesis by altering the expression of oncogenic genes [13]. Recent advances in genome characterization technologies have increasingly enabled the systematic characterization of these alterations in human cancers [14,15]. However, a large number of these identified recurrent somatic copy number alterations (SCNAs) regions usually have no known oncogene or tumor suppressor gene targets (protein-coding genes). This leads researchers to focus their attentions on ncRNAs. Indeed, several studies have identified oncogenic ncRNAs from these recurrent SCNA regions. For example, lncRNA FAL1 is amplified in ovarian cancer, and its small interfering RNAs (siRNAs) can significantly inhibit tumor growth in vivo [16]; the SNORD50A/B snoRNA locus is deleted at a frequency of >10% in each of 12 common cancers, where its loss is associated with reduced survival [17]. This suggests that SCNA is an important feature for estimating the function of ncRNAs.

With the initiation of large-scale cancer genomic projects like The Cancer Genome Atlas (TCGA) [18], the International Cancer Genome Consortium (ICGC) cancer genome projects [19] and the Cancer Cell Line Encyclopedia (CCLE) projects [20], a mass of oncogenomic data has been produced. A number of tools have been created to facilitate the access to multidimensional oncogenomic data and assist with the interpretation of the data, such as Catalogue Of Somatic Mutations In Cancer (COSMIC) [21] and cBioPortal [22,23]. However, these tools mainly focus their attention on protein-coding genes instead of ncRNAs. Although some databases/platforms have also been developed to study the function of ncRNAs [24] such as starBase2.0 [25], lncRNAdb [26], LNCipedia [27] and deepBase2.0 [28] (Supplementary Table 1), they usually ignore the SCNA information of ncRNAs. Thus, a comprehensive catalog for SCNAs of ncRNAs in human cancers is urgently needed.

**Figure 1:**
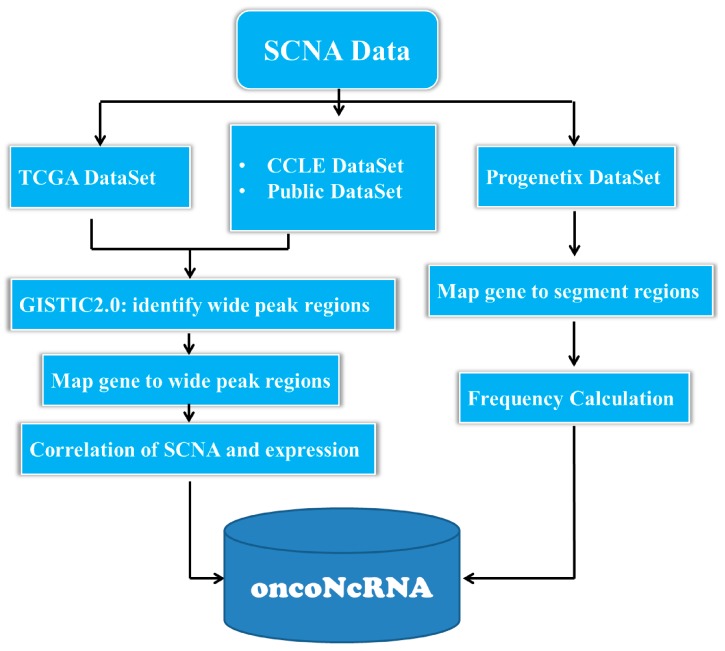
The workflow of oncoNcRNA web portal. We integrated large-scale somatic copy number alteration (SCNA) data from The Cancer Genome Atlas (TCGA), Cancer Cell Line Encyclopedia (CCLE) and other published papers, and provided the correlation of expression level and copy number for over 50,000 genes in 36 TCGA cancer types. Recurrent SCNA regions were identified by GISTIC 2.0 software [29] (Genomic Identification of Significant Targets in Cancer). The oncoNcRNA provided user-friendly web interfaces to query and browse the SCNA information of all well-annotated ncRNAs in up to 64 human cancer types.

In this study, we developed oncoNcRNA to explore the oncogenic potential of ncRNAs from large-scale SCNA data in human cancers. In oncoNcRNA, we performed a large-scale integration of public SCNA data and provided the SCNA information of all well-annotated ncRNAs in up to 64 human cancer types (Figure 1).

Moreover, we also estimated the correlation of expression level and copy number for over 50,000 genes in 36 TCGA cancer types. oncoNcRNA provides user-friendly web interfaces to query and browse the SCNA information of the gene of interest in human cancers. It is expected to help researchers to investigate the potential functions and mechanisms of ncRNAs.

## 2. Results

### 2.1. Identification of ncRNAs with Recurrent SCNAs in Human Cancers

**Figure 2:**
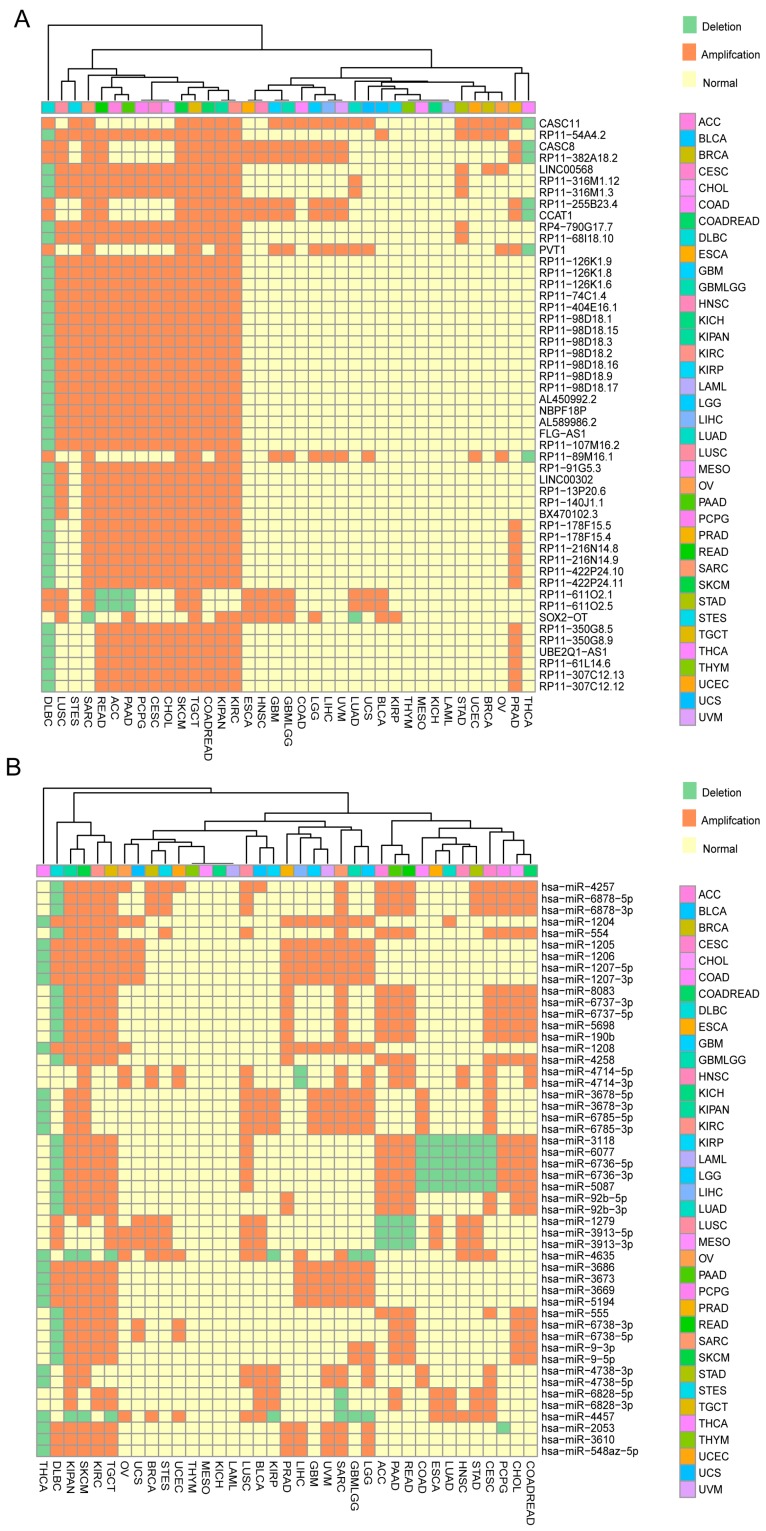
The amplification signatures for the top long non-coding RNAs (lncRNAs) and microRNAs (miRNAs) in the most cancer types. (**A**) The top 50 lncRNAs annotated by GENCODE V19 with the most cancer types for amplification; (**B**) The top 50 miRNAs with the most cancer types for amplification in 37 TCGA tumors.

To help researchers rapidly and conveniently estimate whether a ncRNA has oncogenic roles, we systematically estimated the SCNAs of all well-annotated ncRNAs in 64 human cancers from TCGA [18], Progenetix database [30] and several independent studies [15,20,31]. As a result, we discovered that a larger number of ncRNAs show extensive copy number gain/loss within and across cancer types. For example, for 13,870 lncRNAs annotated in GENCODE version 19, 72.1% (10,001/13,870) of lncRNAs showed significant SCNAs in at least one cancer type for all 37 TCGA cancer types; for 2794 miRNA annotated in mirBase version 20, up to 99.1% (2768/2794) of miRNAs showed significant SCNAs in TCGA tumor cohorts. This finding reconfirms that miRNAs frequently show genomic alterations in human cancers [32,33]. Notably, the majority of ncRNAs possessed SCNAs in at least two cancer types, whereas only a small portion of ncRNAs presented cancer-unique alterations. Figure 2 characterizes the signatures consisting of the top 50 lncRNAs and 50 miRNAs with the most cancer types for amplification. For 50 lncRNAs, cancer susceptibility candidate 11 (CASC11) is ranked the first, while two canonical oncogenic lncRNA, CCAT1 [34,35,36,37,38] and PVT1 [39,40,41], are ranked 9th and 12th respectively. These molecules may be worthy of further experimental investigations.

### 2.2. Estimation of Correlation of ncRNA Gene Copy Number and Expression

**Figure 3:**
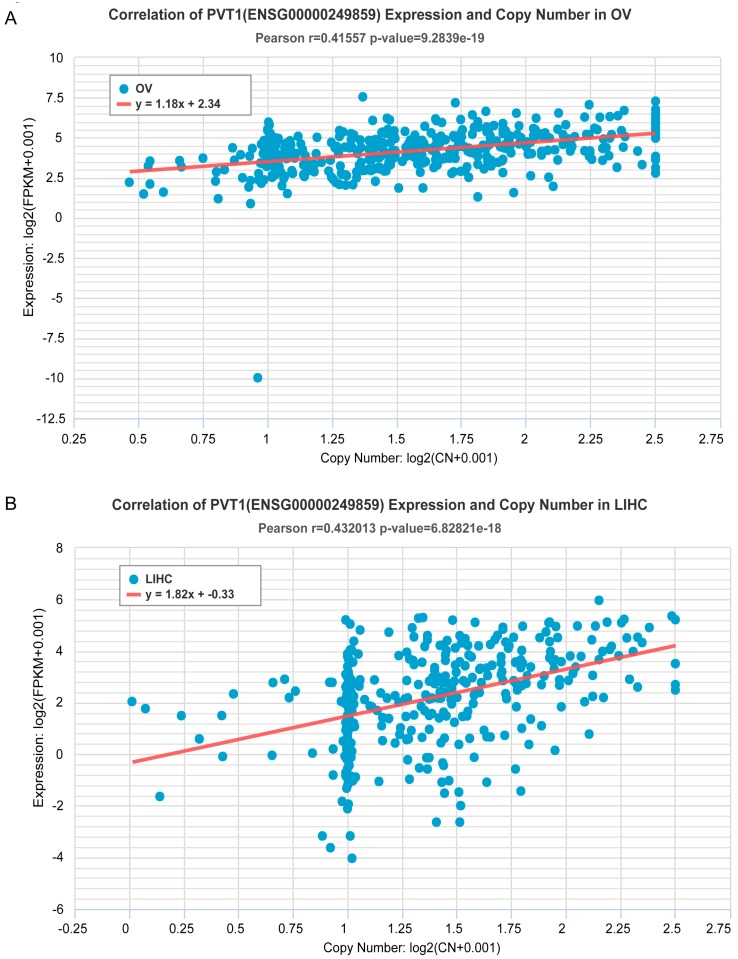
lncRNA PVT1 with significant amplification and overexpression. (**A**) PVT1 shows the significant and positive correlations between expressions and SCNAs in Ovarian Cancer (OV); (**B**) PVT1 shows the significant and positive correlations between expressions and SCNAs in Liver Hepatocellular Carcinoma (LIHC).

Expression alteration is one of the important features to estimate the oncogenic potential of a ncRNA. We speculated that the ncRNA whose expression has a significantly positive correlation with SCNA is more likely to be oncogenic. Therefore, we integrated the available expression values from 36 TCGA cancer types to estimate the 54,312 genes including 1227 miRNAs. This analysis revealed that 54.4% of genes showed significant correlations between expression and copy numbers (Pearson *r* > 0 and *p* < 0.05). The top five genes were protein-coding genes with copy number loss in more than 23 cancer types, including canonical tumor suppressor genes *RB1* [42] and *PTEN* [43]. The top non-coding RNA was found to be a pseudogene named as RPL23AP82, which was deleted in 23 cancer types. Some well-known oncogenic ncRNAs were also included in our results. For example, lncRNA PVT1 [39,40,41] showed significant amplification and overexpression in 12 cancer types. Two of the most significant cancers for lncRNA PVT1 are presented in Figure 3. These data provide useful information for ncRNA studies and clinical biomarker discovery.

### 2.3. The Web-Based Exploration for the Oncogenic Potential of ncRNAs

To help researchers conveniently obtain our analysis results and inquire after a gene of interest, we have developed a user-friendly web portal, oncoNcRNA, for querying and browsing SCNA information for a specific gene. The web portal includes six main modules: lncRNAs, miRNAs, OtherNcRNAs, Proteins, Correlations and Download. The “Download” module allows users to obtain all our analysis results for further investigation.

**Figure 4:**
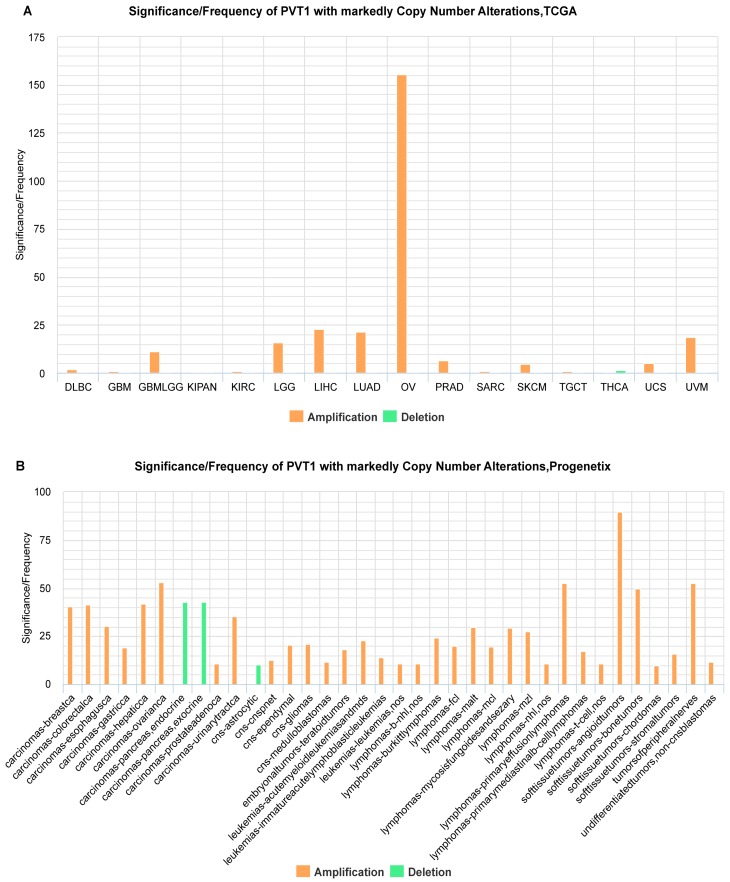
An example showing how users discover the frequently amplified lncRNAs using oncoNcRNA web portal. (**A**) PVT1 shows recurrent SCNAs in 16 TCGA cancer types. The *y* axis shows the –log10 (q value; (**B**) PVT1 shows recurrent SCNAs in 36 Progenetix cancer types. The *y* axis shows the frequency.

According to different gene types, we provide four modules, including lncRNAs, miRNAs, OtherNcRNAs and Proteins, for querying SCNA information about genes in human cancers. With these modules, users can rapidly learn about whether the gene of interest shows recurrent SCNA in a specific cancer type and whether the alteration is extensive across tumor types. Given lncRNA PVT1 for an example, users can rapidly find that PVT1 is frequently amplified in 15 cancer types when selecting the TCGA data cohort through the “lncRNA” module (Figure 4A). When users continue to select other data cohorts, such as the progenetix dataset, PVT1 shows amplification in more than 10% of samples within individual tumors across 33 cancer types (Figure 4B). Users can further obtain the detailed information on genes about cancer types, amplification or deletion, GISTIC 2.0 [29] q value or frequency, residual q value or sample numbers that matched the frequency in result tables when clicking on the gene. The information is also visualized when clicking on the “Frequency” sub-module, and the figure can be exported in multiple formats including PDF file.

The SCNA usually disrupts the expression of genes within it, therefore we estimated the correlation between gene copy number and expression level through the “Correlations” module. With this module, users can easily find whether the expression of the gene of interest is positively correlated with copy numbers in specific cancer type. Meanwhile, users can identify the cancer with the strongest significance for the gene of interest by ranking the correlations and *p* values. When clicking on each cancer type, users can obtain a visual scatter plot which presents the correlation. Through the oncoNcRNA portal, users can rapidly estimate whether the gene of interest has a significant copy number gain/loss within and across tumor types and simultaneously obtain publication-quality figures in a convenient way. A general tutorial is displayed in the “Help and Tutorials” module of website.

## 3. Discussion

With the application of tiling microarrays and high-throughput sequencing technologies into the investigation of whole genomes and transcriptomes, it is now evident that less than 2% of the genome encodes proteins, whereas at least 75% is actively transcribed into ncRNAs [1]. Accumulating evidence has suggested the extensive links between ncRNAs and cancer, such as miRNAs [44,45,46] and lncRNAs [47,48,49]. The most recent study has revealed that the human genome contains over 58,000 lncRNA genes [10], which is far beyond the number of protein-coding genes (~20,000). However, the function of the majority of lncRNAs remains unknown. Despite the fact that some studies have also suggested cancer-related roles of other ncRNAs such as pseudogene [50] and snoRNA [17], our knowledge of the roles of ncRNAs in cancer development remains limited. By integrating large-scale SCNA data from TCGA [18] and other public resources [15,20,30,31], oncoNcRNA reveals a large number of ncRNAs with recurrent SCNAs in human cancers. This may provide a novel insight for the exploration of functional ncRNAs.

oncoNcRNA has unique advantages over other oncogenomic portals, such as Tumorscape [15], COSMIC [21], cBioPortal [22,23] and TANRIC [51]. First of all, oncoNcRNA is mainly designed for exploring the SCNAs of ncRNAs, which is different from other portals with a major focus on protein-coding genes. Second, oncoNcRNAs illustrates the ncRNAs with recurrent SCNAs in tumors. Notably, correlations between gene expressions and copy numbers are also estimated based on available data. Third, oncoNcRNA contains all well-annotated ncRNAs, including miRNAs, tRNAs, snoRNAs and approximately 28,000 novel lncRNAs annotated in mitranscriptome [10]. Finally, oncoNcRNA presents the largest-scale integration of SCNA data for cancer tissues and cell lines from TCGA [18], CCLE [20] and other public resources [15,30,31], and covers up to 64 human cancer types. Taken together, to our knowledge, oncoNcRNA is the first oncogenomic portal for exploring oncogenic potential of all kinds of ncRNAs. It may be a valuable tool for the discovery of functional ncRNAs and clinical biomarker development.

## 4. Materials and Methods

### 4.1. Collecting and Preprocessing the SCNA Datasets in Cancer Genomes

We downloaded the level 4 data of copy numbers of 37 tumor datasets from The Broad Institute TCGA GDAC (release 20150402) [18]. The data from another two large-scale copy number analyses including over 19 tumor types [15,31] and the Cancer Cell Line Encyclopedia (CCLE) [20] were also included. Among these datasets, significantly recurrent SCNA regions were identified by GISTIC 2.0 software [29]. We also downloaded 84 tumor subsets from the type “CLINICALGROUP” on Progenetix [30] and kept 43 of them which contained at least 10 samples for downstream analysis. Progenetix provided the SCNA information of each tumor sample, and used number ”1” to represent amplification while”−1” represented deletion. All genome coordinates were converted to hg19 assemblies by using the UCSC LiftOver Tool [52].

### 4.2. Integrating a Comprehensive Gene Set from Multiple Sources

We integrated the genes annotated by GENCODE (version 19), mitranscriptome [10], mirBase (version 20) [53], piRNABank [54], snoRNABase [55] and GtRNAdb [56]. Mitranscriptome identified over 58,648 lncRNA genes, of which 79% were previously unannotated [10]. We provided detailed information on the isoforms and sequences of each novel lncRNA in oncoNcRNA.

### 4.3. Identification of ncRNAs with Oncogenic Potentials in Human Cancers

We identified the ncRNAs with oncogenic potential by adopting the following way. First, the recurrent SCNA regions, the genomic regions which may contain essential oncogenic drivers, were collected from public resources or identified by ourselves. For the TCGA and several other public datasets (excluding the CCLE datasets and Progenetix dataset), their copy number analyses have been performed and recurrent SCNA regions have been identified by GISTIC 2.0 software (Genomic Identification of Significant Targets in Cancer) [29], therefore we adopted these data for our analysis directly. For the CCLE datasets, only segment copy number data were provided, and thus we performed recurrent SCNA region analysis among all cancer cell lines by using the GISTIC 2.0 software. Then, we detected the ncRNAs within the “wide peak” regions using BEDTools (v2.17.0) [57], since the “wide peak” boundaries have been reported to be most likely to contain the targeted oncogenic drivers [29]. An exception was the Progenetix dataset, which preprocessed segment copy number data with number ”1” for amplification and ”−1” for deletion. This made us unable to identify the recurrent SCNA regions through GISTIC 2.0 software. Therefore, the ncRNAs within these segment DNA copy number alteration regions were directly detected using BEDTools (v2.17.0). Since the ncRNAs with more frequent SCNAs are more likely to act as potential oncogenic drivers, we only selected the genes with higher frequency (>10%) as potential candidates.

### 4.4. Bioinformatics Analysis

The RNA-seq expression values of 36 cancer types from TCGA which were calculated by Toil pipeline were downloaded from the website [58] We extracted samples with matched copy number and expression values for each tumor type using custom Perl script. Then both expression and copy number values were log2 transformed. For ncRNAs with frequent SCNAs identified in the above analyses, we calculated the correlation between gene copy numbers and expression values using the Pearson correlation analysis.

### 4.5. Database and Web Interface Implementation

All data sets were processed and stored in a MySQL Database Management System (v5.7.11). The database query and user interface were developed using PHP and JavaScript. The query result table was based on jQueryUI (v1.11.4) and DataTables (v1.10.7), which is a highly flexible tool for sorting and filtering the search result. The figure was made with JavaScript Highcharts (v4.2.1) library [59], and allows users to export multiple picture formats. The web development was based on Bootstrap (v3.3.5).

## Supplementary Files

Supplementary File 1
