# Supplementary material for: oncoNcRNA: A Web Portal for Exploring the Non-Coding RNAs with Oncogenic Potentials in Human Cancers
Source: Noncoding RNA. 2017 Feb 7;3(1):7. doi: 10.3390/ncrna3010007 (PMC5832004; doi:10.3390/ncrna3010007)
Supplement: Supplementary File 1 [file ncrna-03-00007-s001.pdf]

Supplementary Materials: oncoNcRNA: A Web Portal for Exploring the Non-Coding RNAs with Oncogenic Potentials in Human Cancers

Ze-Lin Wang, Xiao-Qin Zhang, Hui Zhou, Jian-Hua Yang and Liang-Hu Qu

Table S1. ncRNA databases.

Databases	Description	Website	Refs.
**LncRNADisease**	the experimentally supported lncRNA-disease association data	http://www.cuilab.cn/lncrnadisease	[1]
**Lnc2Cancer**	experimentally supported associations between lncRNA and human cancer	http://www.bio-bigdata.com/lnc2cancer/	[2]
**CaSNP**	a comprehensive collection of CNA information from SNP array data	http://cistrome.dfci.harvard.edu/CaSNP/	[3]
**StarBase v2.0**	exploring miRNA-mRNA and miRNA-ncRNA interaction maps from Argonaute and other RBP CLIP-Seq data	http://starbase.sysu.edu.cn/	[4]
**ChIPBase v2.0**	decoding the transcriptional regulatory networks of lncRNAs, miRNAs, other ncRNAs and protein-coding genes from ChIP-seq data	http://rna.sysu.edu.cn/chipbase/	[5]
**deepBase v2.0**	Deeply Exploit Small RNAs, Long non-coding RNAs and Circular RNAs from Deep Sequencing Data	http://rna.sysu.edu.cn/deepBase/	[6]
**lncRNAtor**	a comprehensive resource for functional investigation of long noncoding RNAs	http://lncrnator.ewha.ac.kr/index.htm	[7]
**TANRIC**	An Interactive Open Platform to Explore the Function of lncRNAs in Cancer	http://bioinformatics.mdanderson.org/main/TANRIC:Overview	[8]
**DASHR**	database of small human noncoding RNAs	http://lisanwanglab.org/DASHR	[9]
**lncRNAdb v2.0**	a reference database for long noncoding RNAs	http://www.lncrnadb.org/	[10]
**NPInter v3.0**	noncoding RNA-associated interactions	http://www.bioinfo.org/NPInter/	[11]
**miRCancer**	a microRNA-cancer association database	http://mircancer.ecu.edu/	[12]
**miR2Disease**	a manually curated database for microRNA deregulation in human disease	http://www.miR2Disease.org	[13]

References

1. Chen, G.; Wang, Z.; Wang, D.; Qiu, C.; Liu, M.; Chen, X.; Zhang, Q.; Yan, G.; Cui, Q. LncRNADisease: A database for long-non-coding RNA-associated diseases. *Nucleic Acids Res.* **2013**, *41*, D983–D986.
2. Ning, S.; Zhang, J.; Wang, P.; Zhi, H.; Wang, J.; Liu, Y.; Gao, Y.; Guo, M.; Yue, M.; Wang L.; et al. Lnc2Cancer: A manually curated database of experimentally supported lncRNAs associated with various human cancers. *Nucleic Acids Res.* **2016**, *44*, D980–D985.
3. Cao, Q.; Zhou, M.; Wang, X.; Meyer, C.A.; Zhang, Y.; Chen, Z.; Li, C.; Liu, X.S. CaSNP: A database for interrogating copy number alterations of cancer genome from SNP array data. *Nucleic Acids Res.* **2011**, *39*, D968–D974.
4. Li, J.H.; Liu, S.; Zhou, H.; Qu, L.H.; Yang, J.H. starBase v2.0: Decoding miRNA-ceRNA, miRNA-ncRNA and protein-RNA interaction networks from large-scale CLIP-Seq data. *Nucleic Acids Res.* **2014**, *42*, D92–D97.
5. Zhou, K.R.; Liu, S.; Sun, W.J.; Zheng, L.L.; Zhou, H.; Yang, J.H.; Qu, L.H. ChIPBase v2.0: Decoding transcriptional regulatory networks of non-coding RNAs and protein-coding genes from ChIP-seq data. *Nucleic Acids Res.* **2016**, *45*, D43–D50.
6. Zheng, L.L.; Li, J.H.; Wu, J.; Sun, W.J.; Liu, S.; Wang, Z.L.; Zhou, H.; Yang, J.H.; Qu, L.H. deepBase v2.0: Identification, expression, evolution and function of small RNAs, LncRNAs and circular RNAs from deep-sequencing data. *Nucleic Acids Res.* **2016**, *44*, D196–D202.

7. Park, C.; Yu, N.; Choi, I.; Kim, W.; Lee, S. lncRNAtor: A comprehensive resource for functional investigation of long non-coding RNAs. *Bioinformatics* **2014**, *30*, 2480–2485.
8. Li, J.; Han, L.; Roebuck, P.; Diao, L.; Liu, L.; Yuan, Y.; Weinstein, J.N.; Liang, H. TANRIC: An interactive open platform to explore the function of lncRNAs in cancer. *Cancer Res. 2015*.
9. Leung, Y.Y.; Kuksa, P.P.; Amlie-Wolf, A.; Valladares, O.; Ungar, L.H.; Kannan, S.; Gregory, B.D.; Wang, L.S. DASHR: Database of small human noncoding RNAs. *Nucleic Acids Res.* **2016**, *44*, D216–D222.
10. Quek, X.C.; Thomson, D.W.; Maag, J.L.; Bartonicek, N.; Signal, B.; Clark, M.B.; Gloss, B.S.; Dinger, M.E. lncRNAdb v2.0: Expanding the reference database for functional long noncoding RNAs. *Nucleic Acids Res.* **2015**, *43*, D168–D173.
11. Hao, Y.; Wu, W.; Li, H.; Yuan, J.; Luo, J.; Zhao, Y.; Chen, R. NPInter v3.0: An upgraded database of noncoding RNA-associated interactions. *Database J. Biol. Databases Curation* **2016**.
12. Xie, B.; Ding, Q.; Han, H.; Wu, D. miRCancer: A microRNA-cancer association database constructed by text mining on literature. *Bioinformatics* **2013**, *29*, 638-644.
13. Jiang, Q.; Wang, Y.; Hao, Y.; Juan, L.; Teng, M.; Zhang, X.; Li, M.; Wang, G.; Liu, Y. miR2Disease: A manually curated database for microRNA deregulation in human disease. *Nucleic Acids Res.* **2009**, *37*, D98–D104.
